# Flavonoids Targeting the mTOR Signaling Cascades in Cancer: A Potential Crosstalk in Anti-Breast Cancer Therapy

**DOI:** 10.1155/2022/4831833

**Published:** 2022-06-27

**Authors:** Yaseen Hussain, Haroon Khan, Waqas Alam, Michael Aschner, Abdullah, Khalaf F. Alsharif, Luciano Saso

**Affiliations:** ^1^College of Pharmaceutical Sciences, Soochow University, Suzhou, Jiangsu, China; ^2^Department of Pharmacy, Bashir Institute of Health Sciences, Islamabad, Pakistan; ^3^Department of Pharmacy, Faculty of Chemical and Life Sciences, Abdul Wali Khan University, Mardan 23200, Pakistan; ^4^Department of Molecular Pharmacology, Albert Einstein College of Medicine, Bronx, NY 10461, USA; ^5^Department of Pharmacy, University of Malakand, Chakdara, Dir Lower, Pakistan; ^6^Department of Clinical Laboratory, College of Applied Medical Science, Taif University, P.O. Box 11099, Taif 21944, Saudi Arabia; ^7^Department of Physiology and Pharmacology “Vittorio Erspamer” Sapienza University, 00185 Rome, Italy

## Abstract

Cancer is one of the leading causes of death worldwide. Breast cancer is the second leading cause of death in women, with triple-negative breast cancer being the most lethal and aggressive form. Conventional therapies, such as radiation, surgery, hormonal, immune, gene, and chemotherapy, are widely used, but their therapeutic efficacy is limited due to adverse side effects, toxicities, resistance, recurrence, and therapeutic failure. Many molecules have been identified and investigated as potential therapeutic agents for breast cancer, with a focus on various signaling pathways. Flavonoids are a versatile class of phytochemicals that have been used in cancer treatment to overcome issues with traditional therapies. Cell proliferation, growth, apoptosis, autophagy, and survival are all controlled by mammalian target of rapamycin (mTOR) signaling. Flavonoids target mTOR signaling in breast cancer, and when this signaling pathway is regulated or deregulated, various signaling pathways provide potential therapeutic means. The role of various flavonoids as phytochemicals in targeting mTOR signaling pathways in breast cancer is highlighted in this review.

## 1. Introduction

Breast cancer is the second leading cause of death worldwide [1]. Among breast cancer subtypes, basal breast cancer is characterized by unique stromal–epithelial interactions with distinguishing molecular features [2]. Luminal breast cancers express estrogen and progesterone (PR) receptors and respond to hormone therapies [3]. HR^+^/HER2^–^ correspond to luminal A subtype. HR^+^/HER2^+^ correspond to luminal B subtype. HR^–^/HER2^+^ corresponds to HER2-enriched subtype, whereas HR^–^/HER2^–^ corresponds to triple-negative breast cancer [4]. The HER2-enriched positive breast cancers are characterized by high activation of HER2 and EGFR signaling pathway [5]. In addition, triple-negative breast cancer is a specific and worse subtype of breast cancer that does not express progesterone receptor, estrogen receptor, or HER2 (human epidermal growth factor receptor 2). This cancer is manifested by high metastatic potential, invasiveness, poor prognosis, and proneness to relapse [6]. Treatment and management strategies for breast cancer include surgery, radiation, chemotherapy, hormonal therapy, and gene therapy [7]. At cellular and molecular level, breast cancer is regulated by signaling pathways among which mammalian target of rapamycin (mTOR) is a prominent one. Recent studies have shown that mTOR is a therapeutic target in breast cancer [8]. mTOR inhibitors have been approved for use in postmenopausal women with HR-positive breast cancer [9]. An evidence-based study has shown that exercise inhibits mTOR in triple-negative breast cancer and thus plays a key role in its prevention and control [10]. Targeting this target with therapeutic candidates has shown efficacious outcomes. This signaling pathway is associated with the regulation of proliferation, apoptosis, and autophagy, contributing to anti-breast cancer therapy [11].

Different treatment strategies have been applied for breast cancer [12]. Hormonal therapy for breast cancer treatment has not shown efficacy and faces the problem of reoccurrence. The prominent side effects concerned with hormonal therapy are a challenge and limit the use of this treatment in breast cancer [13]. However, in hormone receptor negative tumor cases, hormonal therapy is not useful and the loss of hormone receptors in recurrent breast cancer poses poor response to hormonal therapy [14]. When breast cancer therapy is resistant to hormonal therapy, it is common to pursue chemotherapy. Similarly, immune and gene therapy is also suffering from problems and challenges that participate poorly in breast cancer therapy. Thus, there is a need for promising strategies that can overcome problems inherent to common breast cancer therapy. Flavonoids—a class of phytochemicals exhibiting phenolic structure in its chemistry—are well known for their beneficial and therapeutic effects in living system. Flavonoids are indispensable nutraceutical components due to their antioxidant, antimutagenic, anticancer, and anti-inflammatory potential. Modulation of cellular enzyme function is a property associated with flavonoids [15].

**Figure 1:**
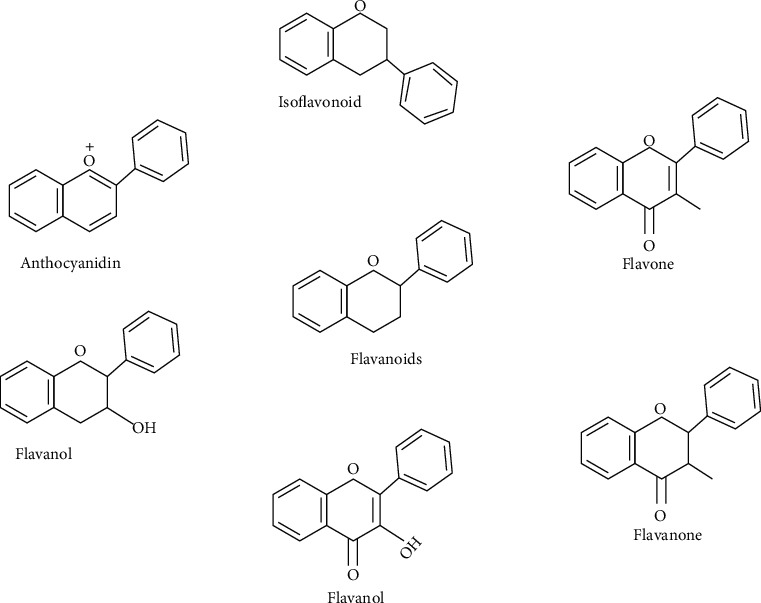
Flavonoids and their chemical structures.

Flavonoid structures are shown in Figure 1. Flavonoid exhibits potent anticancer potential in multiple cancers, such as lung cancer, breast cancer, colorectal cancer, and prostate cancer [16–18]. The mechanism behind its anticancer potential includes its participation in apoptosis induction, autophagy, cell cycle arrest, proliferation, and invasiveness of cancer cells. Also, regulation of cancer-related signaling pathways is a mechanistic route for flavonoids [19].

The mTOR signaling pathway is involved in both extracellular and intracellular signal integration that in turn is responsible for the regulation of proliferation, growth, cell metabolism, and eventually cell survival [20]. It is composed of two complexes—target of rapamycin complexes 1 and 2 (mTORC1 and mTORC2). Cell survival and proliferation are controlled by mTORC2, while metabolism and cell growth are regulated by mTORC1 [21]. The cell growth and tumor development in breast cancer are associated with the mTOR signaling pathway [22].

The regulation and deregulation of the mTOR signaling pathway by flavonoids provide a new insight for its mechanism of action in breast cancer treatment [23]. Triple-negative breast cancer is the most lethal and aggressive form of breast cancer that lacks treatment options. This type of cancer was previously treated with chemotherapy, but unfortunately, resistance develops over time. To cope with this issue, efforts were made to determine the mechanism of chemoresistance by introducing new molecular targets. Among these mechanisms, targeting the mTOR signaling pathway in breast cancer was identified as a potential means for overcoming chemoresistance [24].

Literatures have shown that mTOR is a critical signaling pathway involved in the pathophysiology of breast cancer and flavonoids due to its interfering potential with these mTOR cascades have shown them targeting candidates for breast cancer therapy [25–27]. This review article is aimed at highlighting the role of the mTOR signaling pathway in breast cancer, the potential role of flavonoids in breast cancer therapy targeting the mTOR signaling pathway, and resistant breast cancer as well, exposing mTOR as therapeutic target for flavonoids. Major challenges in the way of breast cancer therapy and their solution through flavonoids were also underpinned.

This article covered all the studies related to flavonoids targeting the mTOR signaling cascades in breast cancer. Up-to-date databases were used for study selection such as PubMed, Google Scholar, Scopus, and Web of Science. The key words searched in our article were “Cancer”, “breast cancer”, “flavonoids”, “mTOR signaling”, and “chemo resistant breast cancer”. The inclusion criterion of the selecting articles was its availability in English because of language issue. A total of 225 articles and 3 book chapters were searched up to the year 2021, of which 125 articles were selected, discussed, and summarized for the provision of a consistent review.

## 2. mTOR Signaling Pathway in Cancer

Mammalian target of rapamycin (mTOR) is a hot target in anticancer therapy that participates in variety of signaling pathways and thus involved in regulation of apoptosis, cell proliferation, and autophagy [28]. Upon activation of the mTOR signaling pathway, it regulates protein synthesis, gene transcription, and tumor metabolism, contributing to immune cell differentiation and regulation of proliferation [29]. Mammalian target of rapamycin (mTOR) is structurally and functionally composed of two complexes, i.e., target of rapamycin complexes 1 and 2 (mTORC1 and mTORC2). Cell survival and proliferation are controlled by mTORC2, while metabolism and cell growth are regulated by mTORC1 [21]. mTOR integrates the stimulation of both signaling pathways that influence protein synthesis and transcription, leading to regulation of autophagy, growth, and apoptosis of cells [30]. The mammalian target of rapamycin (mTOR) signaling pathway is a route for targeting cancers that plays a versatile role in autophagy, cell proliferation, and apoptosis. Different treatment protocols have been reported clinically to have anticancer effect in breast cancer acting via the mTOR pathway Table 1.

Cell division and growth are regulated by the mTOR signaling pathway in normal cells. In cancer cells, mTOR promotes metastasis, growth of tumor cells, and invasion of healthy tissues. During this whole scenario, mTORC1 is activated by associated signaling pathways which along with gene mutation contributes to malignant tumors [41]. The PI3K/AKT/mTOR signaling pathway has a significant regulatory role in cell transcription, translation, and autophagy. Dysregulation of this pathway leads to development, pathogenesis, and prognosis of esophageal cancer; therefore, it is considered as potential target for esophageal cancer therapy and more probably potential target for immunotherapy [42]. The dysregulation of the Wnt/*β*–catenin signaling pathway along with other targets including mTOR leads to certain cancers including pancreatic ductal adenocarcinoma. Targeting this signaling pathway might pose a potential therapeutic strategy for pancreatic cancer. In addition, other targets like melanoma-associated antigen A1 in lung cancer were explored along with its prognostic factors. The expression of melanoma-associated antigen A1 was found high in lung cancer cells [43, 44].

In association, the role of the PI3K/Akt/mTOR signaling pathway was evaluated in colon cancer to characterize survival and proliferation of stem cells. The mentioned signaling pathway (PI3K/Akt/mTOR) significantly controlled colon cancer stem cell proliferation and survival where stem cells were found to cause metastasis and recurrence [45]. Another study found that the mTORC1 signaling pathway was activated by extracellular growth signals from liver kinase B1, which in turn inhibited ring finger protein (RNF 168) activity. The resultant reduction in ubiquitination modification of histone after DNA damage leads to promotion of cancer and malignant cell transformation [46].

**Figure 2:**
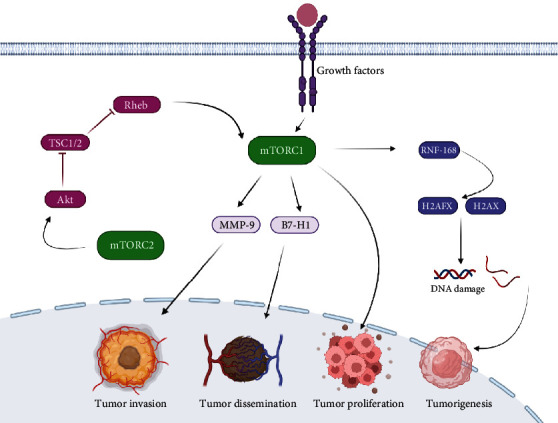
Illustration of mTOR signaling and tumor relationship. Mammalian target of rapamycin (mTORC1) expression is regulated by mTORC2 through Akt/TSC1/2/Rheb signaling pathway that leads to overactivation of mTORC1 and finally results in proliferation and metastasis and encourages tumor formation. In given figure, mutation in cell and extracellular growth signals activate mTORC1 that results in phosphorylation of RNF168 and ubiquitination of H2AFX and H2AX (histone). Overall, the pathway end ups with DNA damage that leads to tumor formation (tumorigenesis). Similarly, mTORC2 activates Akt signaling that promotes binding of Rheb to TSC1/2. It leads to ubiquitination of Rheb, and its activity is reduced. Finally, reduction in Rheb downregulation takes place that triggers mTORC1 activation and inhibits tumor growth. MMP-9 and B7-H1 is upregulated through the mTOR signaling pathway after activation of mTORC1 and results in cancer metastasis and invasion. Abbreviations: mTORC1: mammalian target of rapamycin complex 1; mTORC2: mammalian target of rapamycin complex 2; Akt: serine/threonine-protein kinase; TSC1/2: tuberous sclerosis 1and 2; Rheb: Ras homolog enriched in brain; H2AFX, MMP-9: matrix metalloproteinase–9.

Rheb (a GTPase) has been shown to be involved in mTORC1 activation. In this regard, a reported study indicated that growth factor signals were significantly involved in regulation of Rheb ubiquitination, in turn, inhibiting the expression of the mTORC1 signaling pathway and blocking Rheb activity [47]. The mTORC2/AKT signaling pathway has been shown to be activated by low ubiquitination level of G*β*L, promoting tumorigenesis through activation of oncogenic signaling [48]. Similarly, downstream proliferative cell cycle transcription programs were shown to be activated by promoting mTORC2 activity [49]. In addition, during sufficiency of nutrients, mTOR has been shown to be activated that encourages energy utilization and storage. However, during scarcity of nutrients, for the stability of living system mTOR must be inhibited [50]. The overall relationship between tumor and mTOR is schematically illustrated in Figure 2.

## 3. Challenges in Breast Cancer Treatment

To delay the progression of cancer and to prolong survival are two among the goals of metastasis treatment. Metastatic breast cancer treatment depends upon the site of metastasis, tumor characterization, patient choice, toxicity risk, and comorbidities [51]. Breast cancer can be treated by chemotherapy, surgery, and gene and hormonal therapy [52, 53]. Surgery is one of the common breast cancer treatments that precede chemotherapy and/or hormonal therapy. Surgery is most common in cases of sentinel lymph node and dissection for locoregion [54]. To improve the efficacy of breast cancer treatment, surgery must be used in combination with chemo- or hormonal therapy. However, the associated challenge with surgery in breast cancer therapy involves macromolecular peripheral oxidative damage during the postoperative period [12]. Similarly, radiations are used in breast cancer treatment followed by surgery; however, it poses the challenge of resistance and in patients with 5-year age has shown higher chances of relapse [55]. To cope with the radiation challenge, a recent study is aimed at evaluating the clinical efficacy and side effects of intraoperative radiotherapy after neoadjuvant chemotherapy. A single-arm phase II clinical trial was conducted in 24 breast cancer women in a tertiary referral center in Iran. Results of the study concluded that intraoperative radiotherapy resulted in reduced side effects, cosmetic outcomes, and local control after neoadjuvant chemotherapy in patients with breast cancer [56]. Similarly, such excellent cosmetic outcomes were achieved in this domain in another study by Hosseini et al. [57].

Hormonal therapy is a well-tolerated and effective anti-breast cancer therapy that involves the systemic delivery of hormones specifically in estrogen receptor-positive breast cancer. However, resistance and sensitivity are two main associated problems [14]. Among the systemic delivery of hormone is ovarian suppression that utilizes luteinizing hormone-releasing hormone [58]. Aromatase inhibitors are another choice responsible for estrogen synthesis and thus contribute to metastatic breast cancer treatment. However, it only inhibits aromatase enzyme and androgen circulating level is not affected due to suppression of estrogen. Systemic side effects are also a problem related to such therapy [59]. In case of additive hormonal therapy, the androgen efficacy rate is low and poses certain side effects (hirsutism). Progestin is associated with weight gain [60, 61]. In addition, the epigenetic landscape in breast cancer is prone to alteration through microenvironmental conditions, i.e., hypoxia that leads to convergent disease evolution. More specifically, epigenetic machinery mutation results in transcription and DNA repair defects, which in turn leads to blockade of differentiation, cell death evasion, and tissue invasiveness. To cope with these outcomes is a big challenge in the therapeutic arena of estrogen-positive breast cancer [62]. However, the possible strategies for such challenges include (i) focusing genes involved in estrogen-mediated signaling and ESR1 alterations [63, 64] and (ii) alterations in cell cycle associated with hormone resistant breast cancer and ER*α* regulators [65, 66].

Breast cancer resistant to hormonal therapy is treated by chemotherapy as a first choice. Breast cancer patients, who are at high relapse risk and had received topical therapy, received chemotherapy as an adjuvant treatment [67, 68]. Doxorubicin is a common chemotherapy antibiotic used in the management of metastatic breast cancer, but nausea, vomiting, alopecia, and myelotoxicity limit its use in breast cancer treatment [69]. In case of resistance to doxorubicin and other anthracycline, taxanes have been used as first-line treatment in breast cancer. Combination therapy of taxanes and anthracycline is effective; however, taxanes increase the risk of neuropathy [70]. Due to acquired and intrinsic resistance phenomenon, breast cancer has shown poor response to conventional chemotherapy and in most resistance cases, Akt fingerprint was detected as involved factor [71]. It has been shown that Akt overactivation and its concerned up- and downstream regulators were considered as potential targets for resist anti-breast cancer therapy. Among various targeting approaches, alpelisib was approved as a PI3K/Akt inhibitor for breast cancer therapy [72]. In the context of improving the treatment challenges to therapeutic options in breast cancer, formononetin—Chinese traditional medicine—was used to overcome the everolimus chemoresistance. Results showed that codelivery of everolimus and formononetin reduced tumor volume by twofolds with 22% reduction in cell survival. Everolimus significantly suppressed the mTOR signaling pathway in breast cancer cells [73]. It is concluded that chemoresistance issue to everolimus can be overcome by its codelivery with formononetin for efficient tumoricidal activity in breast cancer therapy.

Poor prognosis and difficulty in treatment of aggressive triple-negative breast cancer are a major challenge in cancer therapy [24]. The cyclin D/cyclin-dependent kinase (CDK) 4/6 pathway is involved in such cell cycle regulating checkpoints. To cope with it, three selective CDK4/6 inhibitors have been approved by the FDA so far for the treatment of triple-negative, estrogen receptor (ER) +/human epidermal growth factor receptor 2 (HER2) breast cancer [74]. In addition, glutamine dependence and Warburg effects are responsible factors for altered metabolism in triple-negative breast cancer. In such scenario, glutamine inhibitor CB839 is under clinical trial and has shown promising therapeutic response. Also, PI3K/Akt/mTOR being an important signaling pathway in breast cancer could be targeted by various therapeutic agents for effective breast cancer therapy and to cope with the faced challenges as well [8]. Immune therapy is another effective choice for breast cancer treatment that involves cytokines, leukocytes, and metagenes that contributes to cancer therapy. Immunotherapy is an effective approach through ablating STAT3 signaling [75]. Despite clinical trials, such therapy has shown minimal clinical efficacy. The skin and gastrointestinal tract suffered due to side effects [76]. The existence of several tumor antigens and aberrant molecular pathways in breast cancer makes it fit candidate for gene therapy. However, toxicity, side effects, and transduction inefficiency related to gene therapy in breast cancer remain a big challenge [77]. Tamoxifen suppresses mTOR and induces apoptosis. Hyperglycemia is a risky factor in the development of resistance in breast cancer because it activates the AKT/mTOR/AMPK signaling pathway and leads to tamoxifen resistance [78]. Cooccurring mutation is another hurdle in breast cancer therapy. A response to platinum-based chemotherapeutics and PARP inhibitors has been predicted due to inactivating mutations in BRCA1/2. However, in multiple tumor types, BRCA1/2 underlie resistance to these therapies. In this regard, the genomic profile with cooccurring BRCA2 and ESR1 mutations in estrogen receptor-positive breast cancer was identified. Olaparib was used as a treatment agent, and the patients showed positive response with long-term benefits [79].

In summary, the existing treatment options for breast cancer treatment exhibit worst consequences and challenges in one way or another. There is a need of an effective treatment option that can overcome these challenges and improve patient survival and life style.

## 4. Flavonoids in Cancer

Over the years, the attention toward the role of phytochemicals in reducing the risk of developing cancer is rising [80]. As a versatile nutraceutical class, flavonoid affords many human-health-friendly benefits. Flavonoids can be used in various ailments and also an effective anticancer agent [19, 81]. In case of lung cancer, flavonoids have shown efficacy via multiple pathways that lead to apoptosis induction, cell cycle arrest, and modulation of carcinogen-metabolizing enzymes [82]. Receptor tyrosine kinase cascade is another target of flavonoids in lung cancer because of its high expression in lung tumor. Cell migration, proliferation, survival, and differentiation are mediated by signal transductions from these cascades. Flavonoids have shown efficacy in interfering with these signal transduction cascades [83]. Similarly, flavonoids combat lung cancer through activation of autophagy and apoptosis resulting [84]. In a recent study, Silymarin—flavonoid extracted from *Silybum marianum*—was investigated for its hepatotoxicity reduction potential in metastatic breast cancer patients treated with doxorubicin/cyclophosphamide-paclitaxel regimen. Results suggested that Silymarin oral administration significantly reduced the hepatotoxicity severity in nonmetastatic breast cancer patients after one-month therapy with the mentioned regimen [85, 86]. Crocin was evaluated in breast cancer chemotherapy characterized by anxiety, toxicity, and depression profile. Results showed that Crocin in breast cancer patients during chemotherapy ameliorated depression and anxiety [87].

Anthocyanidins are a subclass of flavonoids that exhibit anti-colon cancer potential through negative regulation of multiple signaling pathways including signal transducer and activator of transcription (STAT), clear factor kappa light chain enhancer of activated B cells (NF-*κ*B), c-Jun N-terminal kinase (JNK), and mitogen activated protein kinase (MAPK) [88]. Apigenin stimulates apoptosis by increasing p53 expression along with alteration in ratio of Bax/Bcl-2 [89]. Quercetin induces its cytotoxic anti colorectal cancer effect by inhibition of the NF-*κ*B signaling pathway [90]. Epigallocatechin-3-gallate was evaluated in animal models against colorectal cancer, and it was concluded that tumorigenesis was reduced through decrease in expression of Cox2 and the Wnt signaling pathway [91]. Thus, flavonoids play a significant protective role against colorectal cancer following multiple signaling pathways.

Prostate cancer is associated with epigenetics, and any modifications in these epigenetic have a worse relationship with cancer induction. Flavonoids induce epigenetic modifications and in this way deal with prostate cancer. These modifications include long noncoding RNA, microRNA, histone modifications, and DNA methylation [92]. Apigenin, quercetin, silibinin, and genistein have shown effect on epigenetic modifications [93, 94]. In a recent research work, quercetin and luteolin were analyzed for their anticervical cancer potential. Results established reversal of epithelial to mesenchymal transition. Cervical cancer was inhibited through activation of ubiquitin ligase E2S via epithelial to mesenchymal signaling induced by quercetin and luteolin [95]. Thus, ubiquitin ligase E2S could be a possible target for flavonoids in cervical cancer. Similarly, hesperetin-related molecule was isolated from *Cordia sebestena* and evaluated for its anticervical cancer potential. Results indicated a significant anticancer activity in HeLa cells that was revalidated through in silico molecular docking studies. Interaction of cervical carcinoma with E6 protein with significant binding energy was observed in anticervical cancer activity [96]. Resveratrol through inhibition of the Akt (PI3K-Akt) signaling pathway, matrix metalloprotease-9, NF-*κ*B, and protein kinase C provides anticancer effect via antioxidant activity [97].

In summary, flavonoids thus through its multiple mechanisms target various type of cancers (Table 2) and have shown efficacy in cancer treatment. Therefore, flavonoids should be introduced in cancer clinical research to encourage its targeted anticancer potential in patients with aggressive cancer diseases.

## 5. Flavonoids Targeting mTOR in Breast Cancer and Chemoresistance

The progression of cancer is associated with several signaling cascades, and mammalian target of rapamycin (mTOR) is one them. This pathway is involved in triggering malignant transformation [106]. Cell survival, proliferation, metabolism, and cell cycle are mainly regulated by the mTOR signaling pathway. Therefore, flavonoids target such pathway during cancer therapy and pose its anticancer potential [107].

Tangeretin was evaluated for anti-prostate cancer potential targeting the PI3K/Akt/mTOR signaling pathway. Results suggested that following the PI3K/Akt/mTOR signaling pathway, tangeretin effectively induced reprogramming of epithelial to mesenchymal transition and the expression level of mesenchymal proteins in prostate cancer cells was reduced [108]. Fisetin is another flavonoid that contributes its anticancer potential via acting on the Akt/mTOR signaling pathway [109].

Research studying flavonoid molecular docking has probed for an effective inhibitor against mTOR in breast cancer. The designed ligand displayed maximum binding affinity of (Δ*G*—4.91 kcal/mol). In addition, significant efficacy and safety profile was observed [110]. Similarly, eight flavonoids isolated from *Tephroseris kirilowii* were evaluated for breast cancer treatment focusing on the mTOR signaling pathway. The mechanistic approach uncovered for efficacy was associated with inactivation of the Akt and mTOR signaling pathway [111].

Cancer progression and metastasis are associated with cancer stem cells. Li et al. investigated the anti-breast cancer effect of quercetin and its mechanistic insights using MCF-7 breast cancer cell lines. Key findings revealed that after quercetin treatment, MCF-7 cells showed G1 phase arrest and the observed mechanism was inhibition of the PI3K/Akt/mTOR signaling pathway [112]. Fisetin is a flavonoid that targets components of signaling pathways in breast cancer treatment especially those associated with metastatic switches, apoptosis, and cell survival [113]. An *in vivo* orthotopic mammary tumor model (4T1) was analyzed for the anti-breast cancer potential of flavonoid Fisetin. Metastasis, proliferation, and invasiveness of breast cancer cells were significantly suppressed by Fisetin with the induction of apoptosis. Regulation of the PI3K/Akt/mTOR signaling pathway was found to mediate this anti-breast cancer potential [114]. Fisetin is hydrophobic in nature suffering from low bioavailability. Overwhelming such issue with this flavonoid might give fruitful outcome in the future during breast cancer therapy targeting the mTOR signaling pathway.

Flavonoids extracted from *Astragalus membranaceus* i.e., campanulin, ononin, calycosin, and formononetin were evaluated for their anti-breast cancer potential. Breast cancer apoptosis was significantly increased with 25–50 *μ*g/ml concentration of extract as compared to control, whereas the level of mTOR was effectively reduced by the flavonoids [115]. In vivo study in mice showed significant antitumor effect by quercetin at a concentration of 15 mg/kg body weight of mice. In addition, quercetin efficiently inhibited the Akt/mTOR signaling pathway confirmed from western blot results [116]. Lupiwighteone through activation of the Akt/mTOR signaling pathway resulted in apoptosis induction via triggering the caspases and induction of angiogenic activities [117]. Acacetin in breast cancer resulted in autophagy and apoptosis induction trough suppression of the Akt/mTOR signaling pathway [111]. The same results were obtained with Genkwanin [118].

Luteolin is a natural therapeutic candidate for many diseases, and its ability to combat diseases was evaluated against triple-negative breast cancer characterized by proliferation and metastasis. Metastasis and proliferation associated with triple-negative breast cancer were inhibited by luteolin followed by in activation of the Akt/mTOR signaling pathway with significant reversal of epithelial to mesenchymal transition. Overall, regulation of MMP9 expression by luteolin reduced the level of Akt/mTOR. Flavonoids also play a key role in epigenetic regulation during hitting the mTOR signaling pathway as their target [119]. For cell mobility in tumors, glycolysis is the main source of energy supply. Expression of cell migration markers like MMP-2 and MMP-9 was downregulated. In addition, quercetin inactivated the Akt/mTOR pathway and enhanced in autophagy [120].

Medicinal agents with low toxicity and high therapeutic efficacy profile are desirable, and in this regard, flavonoid–metal ion complexes were deliberated. Vanadium–quercetin complex was evaluated against mammary carcinogenesis targeting MCF-7 cell lines. Results of both *in vivo* and *in vitro* studies suggested that formed complex of vanadium upregulated the expression of mTOR, caspases, and p53 in a dose-dependent manner along with induction of apoptosis [121]. Thus, vanadium complex with flavonoids may be a suitable therapeutic candidate for breast cancer. Among flavonoids, Eupafolin extracted from common sage was evaluated for its anti-breast cancer potential. Findings of the study revealed that Eupafolin led to inhibition of cell proliferation in E0771 breast cancer cell lines and caused G0/G1 phase arrest through the PI3K/Akt/mTOR signaling pathway [122]. Flavonoids target the mTOR signaling pathway, contributing to extension of life span [123]. The negative aspects of flavonoids targeting the mTOR signaling pathway are still a challenge that needs further exploration.

**Figure 3:**
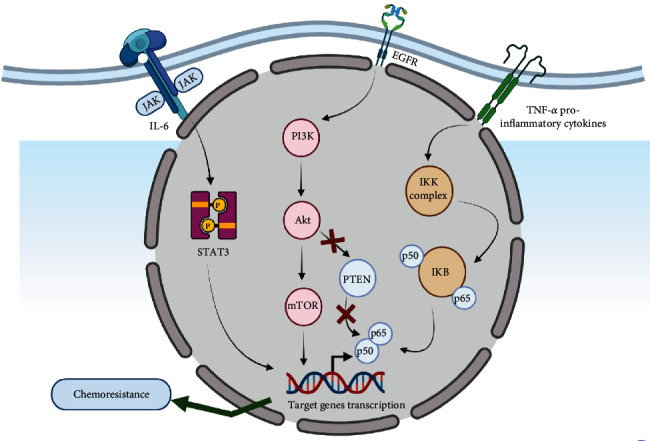
Depiction of mechanistic approaches involved in chemoresistance during breast cancer. Abbreviations: EGFR: epidermal growth factor receptor; IL-6: interleukin–6; TNF-*α*: tissue necrosis factor–alpha; STAT3: signal transducer and activators of transcription 3; JAK2: Janus kinase 2 gene; PI3K: phosphatidylinositol–3–kinase; mTOR: mammalian target of rapamycin; Akt: serine/threonine–protein kinase; PTEN: phosphatase and tensin homolog.

Triple-negative breast cancer is a lethal aggressive form of breast cancer with higher mortality rate. Chemotherapy is the only systemic treatment strategy for triple-negative breast cancer which unfortunately faces the problem of resistance [124]. Certain mechanisms (Figure 3) are involved in chemoresistance of triple-negative breast cancer, and the mTOR signaling pathway is one among these mechanisms. As discussed earlier, the PI3K-AKT-mTORsignaling pathway controls and regulates proliferation, growth, survival, and motility within cellular environment [125]. Phosphatase and tensin homolog (PTEN) is a tumor suppressor that negatively regulates the activity of the PI3K-AKT-mTOR signaling pathway [125].

Triple-negative breast cancer is characterized by hyperactive mTOR signaling, and this hyperactivation is attributed to loss of PTEN in triple-negative breast cancer. In addition, the loss of PTEN is main factor to chemoresistance in breast cancer [126]. A research study reported that inhibition of mTOR signaling significantly sensitizes the chemoresistant cells to anticancer agents in ovarian cancer [127]. It demonstrates that mTOR is a target for therapy, where flavonoids should be considered the targeting agents based on their effect on mTOR signaling. In a research study, the contributing factors toward the disease onset progression and drug response of triple-negative breast cancer were evaluated in Asian individuals. The PI3K–AKT–mTOR signaling pathway and mTOR inhibitors were found interlinked for the treatment of triple-negative breast cancer [128]. Flavonoid targeting the mTOR singling pathway in breast cancer was briefly discussed by Ong et al. [129].

## 6. Discussion

The mTOR signaling pathway is involved in both extracellular and intracellular signal integration that in turn is responsible for the regulation of proliferation, growth, cell metabolism, and eventually cell survival. The regulation and deregulation of the mTOR signaling pathway by flavonoids provide a new insight for its mechanism of action in breast cancer treatment. Triple-negative breast cancer is the most lethal and aggressive form of breast cancer that lacks treatment options. This type of cancer was previously treated with chemotherapy but unfortunately, resistance becomes a big hurdle in the way of chemotherapy. To cope with this issue, efforts were made to determine the mechanism of chemoresistance by introducing new molecular targets. Among these mechanisms, targeting the mTOR signaling pathway in breast cancer was identified as a potent target that overcomes the issue of chemoresistance. However, no sufficient data is available on flavonoids targeting mTOR in chemoresistant breast cancer and thus, it is a future prospect to elaborate the role of flavonoids targeting mTOR in chemoresistant breast cancer. Again, it is worth mentioning that mTOR is a hot target for flavonoids.

## 7. Conclusions

Breast cancer is a big dilemma globally while triple-breast cancer is the most aggressive and lethal form of breast cancer. Conventional therapies for breast cancer offer many drawbacks and challenges, which must be considered to extend the life span of breast cancer patients. Flavonoids were explored for its anticancer potential in many cancers including breast cancer, and it was concluded that through multiple signaling pathways, flavonoids deal with various sorts of cancers. Among these, mTOR signaling was found a potent therapeutic target for flavonoids in breast cancer. Flavonoids through regulation and deregulation of the mTOR signaling pathway control cell growth, proliferation, apoptosis, and autophagy that end up with cell survival. Apart from it, breast cancer is chemoresistant that needs attention. In this regard, flavonoids were targeted in chemoresistant breast cancer focusing on mTOR signaling that resulted in effective therapeutic efficacy. However, inadequate data is available in literature regarding the use of flavonoids in chemoresistant breast cancer that needs further exploration in the future. In addition, clinical trials are also necessary to further validate mTOR as a potential therapeutic target for flavonoids in chemoresistant breast cancer. Then, it will be possible to effectively uplift flavonoids from bench top to markets.

## Figures and Tables

**Table 1:** mTOR inhibitors used in clinical trials for the management of different breast cancers.

Drugs/compounds	Study design	Nature of breast cancer	References
Fulvestrant+everolimus	Phase II clinical trials	Positive breast cancer (estrogen receptor)	[31]
Everolimus	Retrospective	Metastatic breast cancer	[32]
Exemestane+everolimus	Phase III (randomized trial)	Advanced breast cancer (hormone-receptor-positive)	[33]
Temsirolimus	Phase II	Metastatic breast cancer	[34]
Tamoxifen+everolimus	Phase II (randomized trial)	Metastatic breast cancer	[35]
Tamoxifen+sirolimus	Phase I and phase II	HER2-negative breast cancer and hormone receptor-positive	[36]
Plustrastuzumab+vinorelbine+everolimus	Phase III	HER2-positive breast cancer	[37]
Trastuzumab+ridaforolimus	Phase IIb	Trastuzumab-refractory metastatic breast cancer (human epidermal growth factor receptor 2–positive)	[38]
Paclitaxel+trastuzumab+everolimus	Phase II	Advanced breast cancer (HER-2 positive)	[39]
Letrozole+temsirolimus	Phase III randomized	Metastatic breast cancer (hormone receptor-positive)	[40]

**Table 2:** Summary of anticancer mechanism of action of different flavonoids and their results in different cancer cell lines.

Flavonoids	Mechanism of action	Cell lines	Results	References
Quercetin	Quercetin inhibited Akt/PI3 K and MEK-ERK signaling while it augmented UVB-induced nuclear translocation of NF-*κ*b.	Melanoma (B16-F10)	Minimal dosages of quercetin (10–20 M) induce apoptosis in UVB-irradiated melanoma cells via increasing reactive oxygen species (ROS), disrupting calcium homeostasis, and modulating antioxidant defenses	[98]
Apigenin luteolin, resveratrol, and EGC-3-gallate	The investigated compounds cause intracellular copper mobilization and ROS production, resulting in cancer cell death.	Breast cancer (MDA-MB-468), prostate cancer (PC3), pancreatic cancer (BxPC-3)	The investigated compounds cause intracellular copper mobilization and ROS production, resulting in cancer cell death	[99]
Silibinin	Silibinin triggered the MAP2K1/2-MAPK1/3 pathway but blocked the PI3/AKT/mTOR pathway.	Colorectal cancer (SW480)	Silibinin exacerbated oxidative stress in SW480 cells rapidly due to mixed phenotypes of ROS-induced apoptosis and autophagy	[100]
EGC analogs JP8	JP8 causes type I/II cell death in cancer cells by boosting ROS production and activating stress-related proteins like p-eIF2a, IREI, and CHOP.	Melanoma (B16-F10)	In B16-F10 melanoma murine cells, JP8 promotes autophagy and apoptosis but not in normal cells.	[101]
Curcumin (monocarbonyl analogs)	Compound A1 transforms TrxR antioxidant enzymes into a ROS promoter and causes an intracellular ROS explosion. Apoptosis is linked to the formation of reactive oxygen species.	Lung cancer (A549)	Mechanisms of cytotoxicity and proapoptosis	[102]
RWP (red wine polyphenols)	The mechanism of RWP included the suppression of PI3K/Akt kinase signaling, which was independent of its antioxidant potential.	Osteosarcoma (U20s)	RWP caused type I/II mixed cell death in a dose-dependent manner, with the highest effect occurring between 100 and 200 *μ*g/ml equivalents of gallic acid	[103]
Novel synthetic polyphenol conjugate (DPP 23)	In transformed cells, DPP 23 preferentially activates the UPR in the endoplasmic reticulum via ROS production and caspase-dependent death.	Glioblastoma, pancreatic, beast, hepatocellular cancer cell lines	DPP 23 causes cancer cell lines to produce more ROS and activate apoptosis while having no impact on healthy cells	[104]
Tetrahydroxy-trans-stilbene derivatives	Oxidative damage, reduction of mRNA expression and superoxide dismutase activity, reduction of mitochondrial capacity, and glutathione depletion were all associated with cell death.	T cell leukemia (Jurkat cells)	Enhanced caspase 3 and 9 expression and cytotoxic activities	[105]
